# Sterol carrier protein-x gene and effects of sterol carrier protein-2 inhibitors on lipid uptake in *Manduca sexta*

**DOI:** 10.1186/1472-6793-10-9

**Published:** 2010-06-09

**Authors:** Min-Sik Kim, Que Lan

**Affiliations:** 1 Division of Pharmaceutical Sciences, School of Pharmacy, University of Wisconsin, 777 Highland Avenue, Madison, WI 53705-2222, USA; 2 Department of Entomology, University of Wisconsin, 1630 Linden Drive, Madison, WI 53706, USA

## Abstract

**Background:**

Cholesterol uptake and transportation during the feeding larval stages are critical processes in insects because they are auxotrophic for exogenous (dietary) cholesterol. The midgut is the main site for cholesterol uptake in many insects. However, the molecular mechanism by which dietary cholesterol is digested and absorbed within the midgut and then released into the hemolymph for transportation to utilization or storage sites is poorly understood. Sterol carrier proteins (SCP), non-specific lipid transfer proteins, have been speculated to be involved in intracellular cholesterol transfer and metabolism in vertebrates. Based on the high degree of homology in the conserved sterol transfer domain to rat and human SCP-2, it is supposed that insect SCP-2 has a parallel function to vertebrate SCP-2.

**Results:**

We identified the *Manduca sexta *sterol carrier protein-x and the sterol carrier protein-2 (MsSCP-x/SCP-2) gene from the larval fat body and the midgut cDNAs. The MsSCP-x/SCP-2 protein has a high degree of homology in the SCP-2 domain to other insects' SCP-2. Transcripts of MsSCP-2 were detected at high levels in the midgut and the fat body of *M. sexta *during the larval stages. Recombinant MsSCP-2 bound to NBD-cholesterol with high affinity, which was suppressed by sterol carrier protein-2 inhibitors.

**Conclusions:**

The results suggest that MsSCP-2 may function as a lipid carrier protein *in vivo*, and targeting insect SCP-2 may be a viable approach for the development of new insecticides.

## Background

In insects, cholesterol is required as a structural component of cellular membranes and as a precursor for the insect molting hormone (ecdysone). However, insects lack several enzymes in the cholesterol biosynthesis pathway [1], which makes them auxotrophic for exogenous (dietary) cholesterol for satisfactory growth, development and reproduction [2-4]. Therefore, cholesterol uptake and transportation are critical for their survival. Uptake of cholesterol involves intracellular transfer and intercellular transportation. Intracellular cholesterol transfer enables the redistribution of cholesterol within a cell, and intercellular transport is needed for the distribution of cholesterol between cells. It is very well documented that lipophorin (Lp) and lipid transport particles (LTPs) are responsible for the intercellular transportation of cholesterol and lipids in insects [5-7].

Cholesterol is highly hydrophobic with solubility of ~10 nM in water at room temperature [8], can be easily inserted into the lipid bilayer and has a fast flip-flop rate (t_1/2 _= 1-2 minutes) within the lipid bilayer [9]. Diffusion of cholesterol into the lipid bilayer can happen readily, and an aqueous diffusion model has been proposed to account for some of the cholesterol uptake by cells [10]. However, the desorption of membrane-bound cholesterol is slow without the aid of a carrier protein [10,11] to enhance the desorption of membrane-bound cholesterol through the collision with membrane-bound cholesterol [12]. In vertebrates, sterol carrier protein-2 increases the rate of cholesterol transfer between membrane structures from t_1/2 _= 4 days to t_1/2 _= 7-15 minutes *in vitro *[11], demonstrating the potential of lipid carrier proteins to enhance cellular lipid uptake.

Insect cells do not transfer cholesterol between the midgut and the fat body via receptor mediated endocytosis of Lp. The diffusion of cholesterol from Lp to target cell membranes is thought to be the main mechanism for the transport of cholesterol to cells [13]. Moreover, Lp is the only apolipoprotein found in insects and Lp is not synthesized in the midgut [14,15], which excludes the possibility of efflux of absorbed lipids from the midgut into the hemolymph via exocytosis of apolipoprotein/lipid vehicles [7,16]. Therefore, nonvesicular transport of intracellular cholesterol may be important in insect cellular cholesterol uptake via intracellular sterol carrier protein(s) to aid the desorption of membrane-bound cholesterol and the delivery of the cholesterol within the cytosol.

Little is known about the protein(s) involved in the intracellular transfer of cholesterol in insects. Several insect intracellular sterol carrier protein genes have been identified such as the sterol carrier protein-x [17-19], the sterol carrier protein-2 [20], and the steroid acute regulator protein (StAR)-related lipid transfer protein [21,22]. In most cases, the function of those insect intracellular sterol carrier proteins is deduced from the temporal and spatial expression profiles and from the known function of their vertebrate homologs.

Genomic sequence data show that insects may have multiple copies of sterol carrier protein-2 (SCP-2)-like proteins [18,23,24], in contrast to the single copy of the SCP-x/SCP-2 gene in vertebrates [25,26] and in the silkworm, *B. mori *[27]. SCP-x belongs to the SCP-2 gene family, which includes SCP-2, SCP-x, 17β-hydroxysteroid dehydrogenase IV, UNC-24, Metallo-β-lactomase and stomatin [26,28]. In vertebrates, the SCP-x/SCP-2 gene has two initiation sites for coding proteins. One site codes for SCP-x which is post-translationally cleaved into 2/3-oxoacyl-CoA thiolase and SCP-2. A second site codes for SCP-2. Both SCP-x and SCP-2 share a common SCP-2 C-terminus. Increasingly, data suggest that 3-oxoacyl-CoA thiolase is involved in the oxidation of branched chain fatty acids and SCP-2 is involved in the transfer of lipids [29,30]. Whether insect SCP-2 and SCP-2-like proteins have similar or divergent functions is unknown. Some insect SCP-2 and SCP-2-like proteins lack the peroxisome targeting sequence at the C-termini, suggesting that they are cytosolic proteins [23,31]. AeSCP-2, an *Aedes aegypti *SCP-2 protein which is not produced from the SCP-x gene, binds to both cholesterol [20] and fatty acid [32], which is consistent with the broad spectrum of lipid ligands of the vertebrate SCP-2. Over-expression or knockdown of AeSCP-2 gene expression affects cholesterol uptake *in vivo *[23,31,33]. Chemical inhibitors of AeSCP-2 suppress cholesterol uptake in cultured cells from *Aedes aegypti *and *Manduca sexta*, indicating that *M. sexta *has a protein homologous to AeSCP-2 [34]. The tobacco hornworm, *Manduca sexta*, has been one of the primary experimental insect model systems for sterol metabolism studies [35]. Studies using *M. sexta *have contributed to a broad picture of lipid metabolism and have revealed that cholesterol is transported from the midgut to the fat body via lipophorin (Lp) in the hemolymph in the free form, and absorbed cholesterol is stored as cholesterol este

Here, we report molecular cloning and characterization of cDNA encoding SCP-x and SCP-2 from the tobacco hornworm (*Manduca sexta*), and the effect of sterol carrier protein-2 inhibitors (SCPIs) on cholesterol and fatty acid uptake in *M. sexta *larvae. The results suggest that MsSCP-x/SCP-2 is involved in cholesterol absorption and transportation in insects.

## Results

### Molecular cloning and nucleotide sequence analysis of MsSCP-x/SCP-2

**Figure 1:**
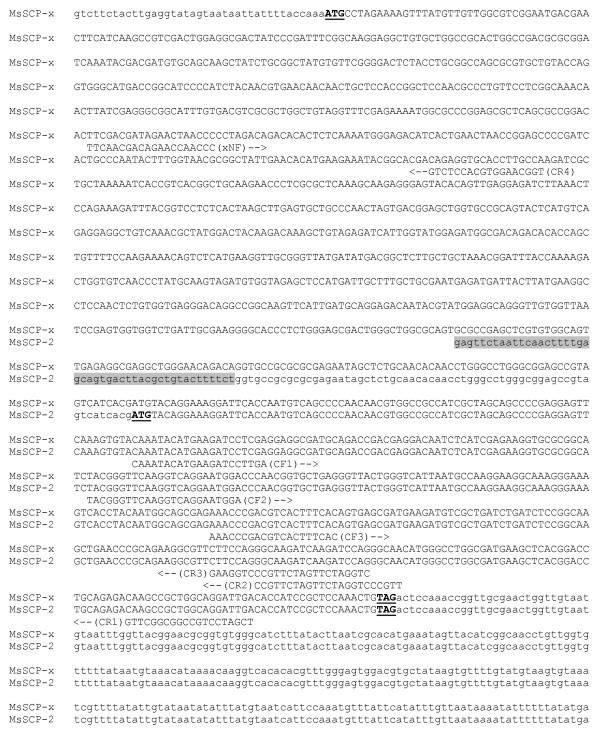
**The nucleotide sequences of MsSCP-x (GQ869536) and MsSCP-2 (GQ869537) cDNAs**. The start and stop codons are bold/underlined. The divergent 5' UTR sequence in MsSCP-2 is in shaded in gray color. Potential intron/exon splicing site in the MsSCP-2 5'UTR is in bold lower case letter. Sequences of forward and reverse primers used in cloning and RT-PCR are aligned under the cDNA sequence with arrows indicating the orientation for PCR reactions.

A PCR-based cloning strategy was used to clone the MsSCP-x/SCP-2 gene, using cDNAs from the larval midgut and fat body as templates. Degenerate primers (MsSCP-CF1 and MsSCP-CR1) allowed us to obtain a cDNA fragment highly homologous to the SCP-2 domain of BmSCP-x and SlSCP-2 (Table 1). The degenerated primer xNF and gene specific primer CR2 produced a cDNA fragment of about 1100 bp, which matches the 2/3-oxoacyl-CoA thiolase domain of insect SCP-x. The 3'-RACE using MsSCP-CF3 primer (Fig. 1) produced independent cDNA clones of about 550 bp containing a putative translation stop site (Fig. 1). Additional gene specific primers (MsSCP-CR4: 5'-TGG CAA GGT GCA CCT CTG-3' and MsSCP-CR3: 5'-AGA ACT AGA ACG GGA CCT TC-3') derived from partial MsSCP-x cDNA sequence were synthesized and used for the 5'-RACE to obtain the 5'-ends of the cDNA. The 5'-RACE using CR4 primer produced cDNA clones of 553 bp containing a putative translation start site at position 39 (Fig. 1). All results taken together indicate that a putative full-length MsSCP-x/SCP-2 gene spans a total of 1918 bps and encodes a protein of 536 amino acids (GQ869536). Interestingly, 6 independent cDNA clones from the CR3 primer 5'-RACE had 46 bp sequences at the 5'-end divergent from the MsSCP-x (Fig. 1 gray colored shaded sequence), suggesting that the MsSCP-2 transcript (GQ869537) may be made from alternative splicing or an alternative initiation site. The deduced amino acid sequence of the coding region showed high similarity to members of the SCP-2 gene family in other insect species (Table 1). The SCP-2 domain of *Manduca *SCP-x has amino acid sequence identities of 97%, 93%, 79%, 69%, and 69% with *Bombyx mori, Spodoptera littoralis, Aedes aegypti, Apis melifera*, and *Drosophila melanogaster*, respectively (Table 1). The MsSCP-x/SCP-2 gene encodes the SCP-x protein with much higher homology to BmSCP-x and SlSCP-x than the Dipteran SCP-x such as AeSCP-x and DmSCP-x (Table 1).

**Table 1:** Identities of deduced amino acid sequences of the MsSCP-x/SCP-2 (Accession# GQ869536 and GQ869537) to the proteins of insect SCP-x gene in other insect species

	SCP-x	2/3-oxoacyl-CoA-thiolase domain	SCP-2 domain
*Bombyx mori*	91.79%	90.48%	96.55%
*Spodoptera littoralis*	89.37%	88.33%	93.10%
*Drosophila melanogaster*	61.88%	60.57%	68.64%
*Apis melifera*	60.81%	59.29%	68.97%
*Aedes aegypti*	64.51%	60.71%	78.45%
*Aedes aegypti (AeSCP-2)*			23.47%

### Tissue and stage expression profiles of MsSCP-x/SCP-2

**Figure 2:**
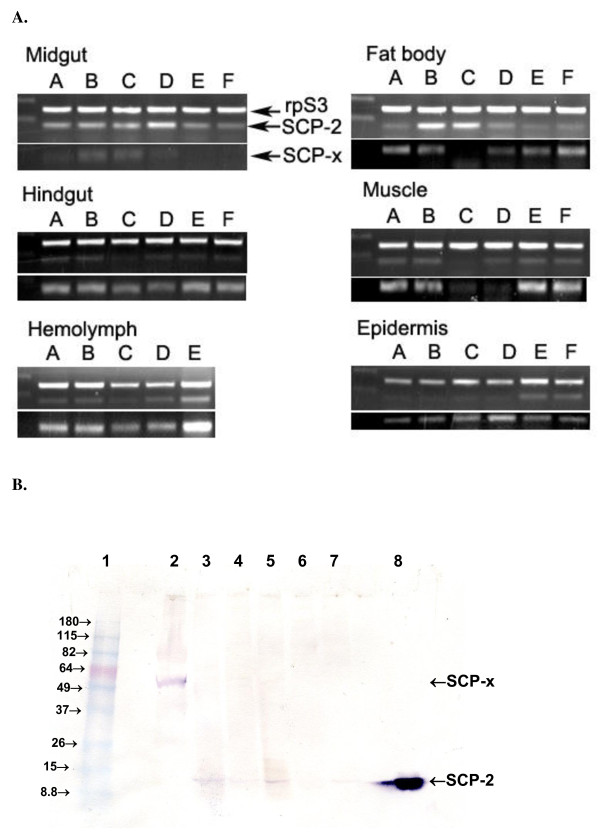
**Expression of MsSCP-x and MsSCP-2**. (A) The semi-quantitative RT-PCR shows that mRNA levels from different tissues at different developmental stages of *M. sexta*. Lane A: Day 1-4^th ^instar; lane B: Day 3-4^th ^instar; lane C: Day 1-5^th ^instar; lane D: Day 4-5^th ^instar; lane E: Day 1-wandering stage; lane F: Day 1-pupal stage. The *Manduca *rpS3 gene was used as internal standard for the RT-PCR. Only 30 cycles of PCR were performed to avoid saturating the signals. (B). Proteins from different tissues of Day 3-4^th ^instar *M. sexta *larva were loaded 20 μg per lane except lane 8 that only had 10 ng purified recombinant MsSCP-2. Protein was analyzed on 4-20% gradient SDS PAGE. Lane 1: protein size marker; Lane 2: hemolymph; Lane 3: midgut; Lane 4: hindgut; Lane 5: fat body; Lane 6: muscle; Lane 7: epidermis; Lane 8: recombinant MsSCP-2.

The transcription profiles of MsSCP-2 in different tissues and stages were determined by semi-quantitative RT-PCR. The MsSCP-2 mRNA level was significantly higher in the midgut and fat body in feeding larvae (Fig. 2A, upper panel for midgut lanes A-D). MsSCP-2 was strongly expressed throughout the examined larval stages and decreased slightly in the wandering and pupal stages (Fig. 2A, upper panel for midgut lane E-F). In the fat body, there was a transient increase in MsSCP-2 transcripts at the end and beginning of each instar (Fig. 2A, upper panel for fat body lanes B-C). Low levels of MsSCP-2 mRNA were detected in the hemolymph, hindgut, epidermis and muscle (Fig. 2A). However, primers corresponding to the 2/3-oxoacyl-CoA thiolase domain detected MsSCP-x mRNA at moderate and high levels in all tissues except the midgut (Fig. 2A, lower panel for midgut lanes A-F). Data from RT-PCRs showed that the transcription of SCP-x and SCP-2 mRNAs from the MsSCP-x/SCP-2 gene is regulated differently in different tissues. MsSCP-2 mRNA was the predominant transcript from this gene in the midgut during the feeding stage (Fig. 2A, midgut, lanes A-D). In the midgut, the levels of MsSCP-2 transcript increased during the 5^th ^larval instar without any increase in the level of MsSCP-x transcript (Fig. 2A, midgut, lanes C-D). This result implies that the MsSCP-x/SCP-2 gene might produce two transcripts: SCP-x mRNA and SCP-2 mRNA, which is similar to the human SCP-x/SCP-2 gene and other lepidopteran SCP-x/SCP-2, BmSCP-x/BmSCP-2 [26,27].

**Figure 3:**
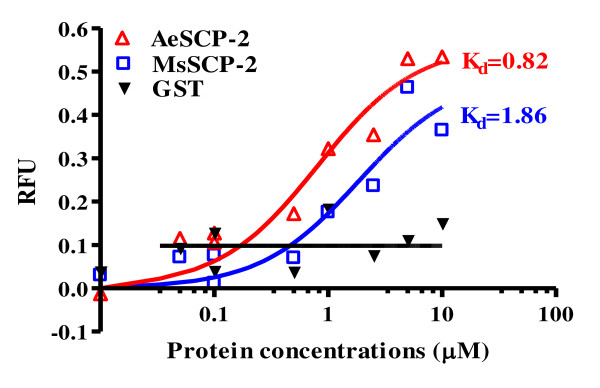
**NBD-cholesterol binding assays for MsSCP-2, AeSCP-2 and GST**. The data were processed using PRISM4.0 software. RFU = Relative fluorescence unit.

Western blotting analysis was performed to investigate the existence of an SCP-2 homolog in tissues from Day 3 4^th ^instars using affinity-purified anti-SCP-2 domain polyclonal antibodies of mosquito SCP-x, AeSCP-x [31]. Figure 3 shows that a protein similar to the molecular weight of 14 kDa (the predicated molecular weight of MsSCP-2 is 14,134 Da) was detected by anti-AeSCP-x antibodies, suggesting that *M. sexta *may have a SCP-2 analog. Tissues expressing MsSCP-2 in *M. sexta *at high levels were the midgut and fat body, which was consistent with the results of RT-PCR (Fig. 2A, lane B). Trace amounts of SCP-2 were also detected in the hindgut and epidermis (Fig. 2B, lane 4 and 7). A band of about 58 kDa protein in the hemolymph was detected by the anti-SCP-2 domain antibody, which was presumably the full-length MsSCP-x. In both the hindgut and epidermis, the predominant transcript from the MsSCP-x/SCP-2 gene was the SCP-x mRNA (Fig. 2A, lane B), whereas the MsSCP-2 protein was the only detectable product of the MsSCP-x/2 gene (Fig. 2B, lanes 4 and 7). The results indicate that the full length MsSCP-x protein was proteolytically cleaved to produce the SCP-2 domain protein in those tissues. The MsSCP-x protein was not processed to produce MsSCP-2 in the hemocytes due to the prominent SCP-x band and the lack of the MsSCP-2 band (Fig. 2B, lane 2).

### Ligand and SCPIs binding in MsSCP-2

**Figure 4:**
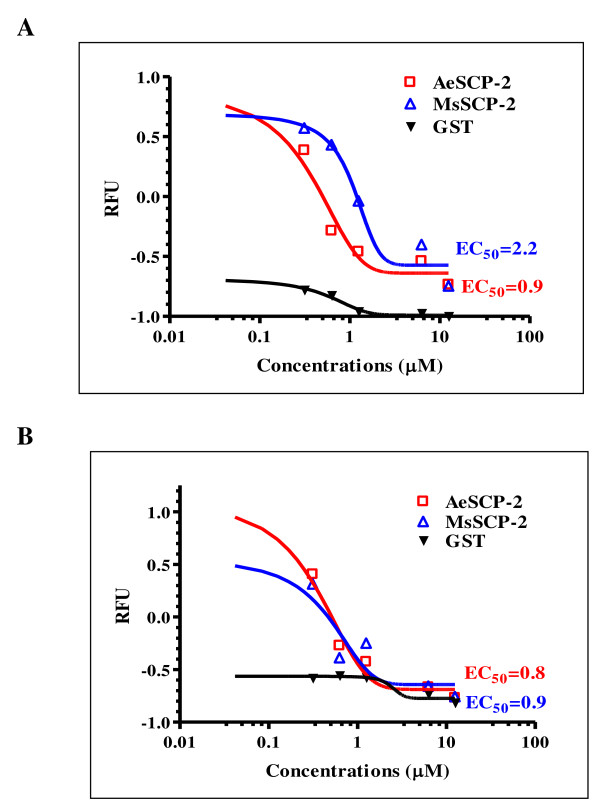
**NBD-cholesterol and SCPIs competition binding assays to MsSCP-2**. Assays for NBD-cholesterol and sterol carrier protein-2 inhibitors (SCPIs; A: SCPI-1; B: SCPI-2) competitive binding to MsSCP-2. The background NBD-cholesterol fluorescence (NBD-cholesterol alone in the reaction buffer) was deducted from each assay. Shown are net changes in NBD-cholesterol fluorescence in intensity (RFU = Relative fluorescence unit) in the presence of increasing concentrations of each SCPI. GST and AeSCP-2 were used as negative and positive controls, respectively. The data were processed using PRISM4.0 software.

The binding affinity (*K*_*d*_) of recombinant MsSCP-2 to the cholesterol analog was determined using an NBD-cholesterol binding assay. As shown in Figure 4, increasing the concentrations of MsSCP-2 (over the range 1 nM~10 μM) while maintaining the concentration of NBD-cholesterol constant (1 μM) resulted in increased fluorescence emission of NBD-cholesterol. Analysis of the binding curve using the one site binding non-linear regression model in GraphPad PRISM software (Ver. 4.0) yielded high binding affinity for both MsSCP-2 and AeSCP-2 (positive control). *Aedes aegypti *SCP-2 (AeSCP-2) is not transcribed from the SCP-x gene [18] but belongs to the SCP-2 gene family, and has been shown to have high affinity to cholesterol [20]. The binding affinity (*K*_*d*_) was 1.86 × 10^-6 ^M (R^2 ^= 0.9039) for MsSCP-2 and 8.2 × 10^-7 ^M (R^2 ^= 0.9231) for AeSCP-2, respectively. However, the GST (negative control) showed little binding to NBD-cholesterol (Fig. 3, GST). Although Scatchard plots are useful for visualizing data, they are not the most accurate estimation. In this experiment, we used the one site binding non-linear regression model, since the linear transformation of Scatchard plots distorts the experimental error.

The inhibitory effect of SCPIs on cholesterol binding to MsSCP-2 was measured using NBD-cholesterol competition assays. SCPI-1 and SCPI-2 reduced NBD-cholesterol binding to MsSCP-2 in a concentration-dependent fashion (Fig. 4). AeSCP-2 and GST proteins were used as positive and negative controls, respectively. SCPIs inhibited the binding of NBD-cholesterol to both MsSCP-2 and AeSCP-2. However, SCPIs showed no effect on the binding of NBD-cholesterol and GST, which is a non-binding protein to lipid species (Fig. 4). The 50% maximal effective concentration (EC_50_) of SCPI-1 and SCPI-2 to block NBD-cholesterol-MsSCP-2 binding was determined using the one site competition binding, non-linear regression model in GraphPad PRISM^® ^software version 4.0 (GraphPad Software Inc., San Diego, CA). The EC_50_s of SCPI-1 were 2.2 μM (R^2 ^= 0.9531) and 0.9 μM (R^2 ^= 0.9801) for MsSCP-2 and AeSCP-2, respectively (Fig. 4A). The EC_50_s of SCPI-2 were 0.9 μM (R^2 ^= 0.8832) and 0.8 μM (R^2 ^= 0.9773) for MsSCP-2 and AeSCP-2, respectively (Fig. 4B). The result suggests that SCPIs have high binding affinities to insect SCP-2s and are able to compete with NBD-cholesterol for binding to MsSCP-2 and AeSCP-2. The result also indicates that SCPI-2 may be more effective in inhibiting MsSCP-2's function than that of SCPI-1 (Fig. 4), which is consistent with the observation in the mosquito AeSCP-2 [34]. Whether the differences in the EC_50 _values of SCPI-1 and SCPI-2 in inhibiting cholesterol binding to MsSCP-2 result in different biological effects will be described later.

### Effects of SCPIs on lipid uptake in *M. sexta*

To determine whether MsSCP-2 is involved in cholesterol uptake *in vivo*, we investigated the effects of SCPIs on cholesterol absorption in gate II day 3 4^th ^instar larvae when MsSCP-2 expression was high in both the midgut and fat body (Fig. 2A, lane B). Larvae were fasted for 2 hours, then were fed 0.1 g of diet containing [1,2-^3^H]-cholesterol and SCPI-1 and SCPI-2 at LD_50 _dosages [34]. Tracking the ingested [1,2-^3^H (N)]-cholesterol in *Schistocerca gregaria *showed that insects appear not to alter the ^3^H label on the [1,2-^3^H]-cholesterol *in vivo*, therefore, measuring the amount of [1,2-^3^H]-cholesterol can be used directly as an indication of cholesterol uptake [36]. Moreover, *Manduca sexta *larvae absorb free [^3^H]-cholesterol readily with little modification of the absorbed [1,2-^3^H]-cholesterol [37].

**Figure 5:**
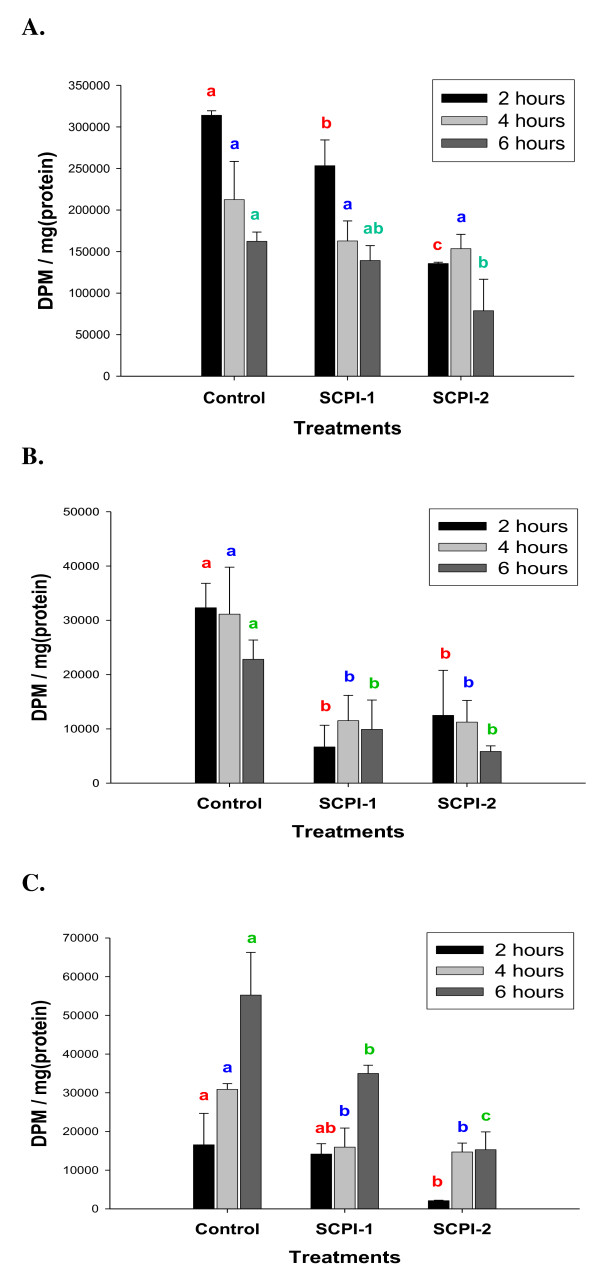
**Effects of SCPIs on [1,2-^3^H (N)]-cholesterol uptake in *M. sexta *Day 3 4^th ^instar larvae**. **(A) **The midgut. **(B) **The hemolymph. **(C) **The fat body. Values = mean ± S.D. (N = 2). Details of the label/chase experiment are provided in the "Materials and Methods". The same letters above the bars in each time point represent that the mean values did not differ from other group of the same time point significantly (*p *> 0.05) in paired t-tests.

The lipophorin in the hemolymph uploads absorbed dietary lipids at the basal side of the midgut and transports the cholesterol to the fat body for storage [14,16]. It was hypothesized that cytosolic lipid transport proteins in the midgut transfer absorbed dietary lipids toward the basal side of the epithelium where lipophorin picks up the lipids [7,16]. We speculated that MsSCP-2 could be one of the midgut sterol carrier proteins that aid the uptake of cholesterol because MsSCP-2 was expressed at high levels in the midgut and fat body (Fig. 2A and 2B) and MsSCP-2 bound to a cholesterol analog (Fig. 2B). To determine whether the inhibition of MsSCP-2 function would affect cholesterol uptake from the diet into the midgut,  [^3^H]-cholesterol in midgut tissue was measured at different time points after feeding. In the midgut tissue, [^3^H]-cholesterol content decreased significantly in the controls between 2 and 6 hours post feeding (*p *= 0.003), indicating the clearance of absorbed [^3^H]-cholesterol over time from the midgut tissue (Fig. 5A, control). The result is consistent with the previous report of a slow but steady clearance of [^3^H]-cholesterol from the midgut within 6 hours post feeding [37]. In SCPI-1 treated larvae at the 2 hour time point, the total [^3^H]-cholesterol in the midgut tissue was slightly lower than that of the control group, whereas, the SCPI-2-treated group had significantly much less (*p *= 0.01; 43.18% of the controls) labeled cholesterol (Fig. 5A, 2 hour time point). At 6 hours post feeding, there was a significant decrease of [^3^H]-cholesterol in the midgut in both the control and SCPI-1-treated groups (*p *= 0.04), indicating that absorbed cholesterol was likely transported out of the midgut tissues (Fig. 5A, 4 hour time point). There was no significant change in the levels of labeled cholesterol 2 to 6 hours post feeding in SCPI-2-treated groups (Fig. 5A, 4 hour time point), which suggests that there was little transfer of absorbed cholesterol. Over the observed time period, the total amount of [^3^H]-cholesterol in the midgut tissue was significantly lower in SCPI-1 and SCPI-2-treated larvae than in the controls (*F*_1,6 _= 8.627, *p *= 0.026 and *F*_1,6 _= 51.47, *p *= 0.0004, respectively). The amount of [^3^H]-cholesterol in the midgut 2 to 6 hours post feeding was 688640.7, 555098.9, and 367836.9 DMP/mg protein for the control, SCPI-1-, and SCPI-2-treated groups, respectively (Fig. 5A). The results imply that the uptake of [^3^H]-cholesterol into the midgut tissue in SCPI-treated larvae was less efficient than in the controls. SCPI-2 was more effective in inhibiting cholesterol binding to MsSCP-2 than that of SCPI-1 (Fig. 4). SCPI-2 had severer effects on MsSCP-2's function *in vivo *resulting in a 46% greater reduction in [^3^H]-cholesterol uptake/transport than the SCPI-1-treatment (*F*_1,6 _= 19.55, *p *= 0.0045) in the midgut tissue (Fig 5A, SCPI-1 and SCPI-2).

To determine whether the inhibition of MsSCP-2 function would affect cholesterol transfer from the midgut to the hemolymph, [^3^H]-cholesterol in the hemolymph was measured at different time points after the feeding. In the hemolymph, constant high levels of [^3^H]-cholesterol were observed over the time period in the control group (Fig. 5B), which is consistent with the observation that hemolymph [^3^H]-cholesterol levels in the label/chase experiments remains steady 4-18 hours post feeding [38]. However, the hemolymph [^3^H]-cholesterol levels were significantly lower (*F*_1,6 _= 38.72, *p *= 0.0008 and *F*_1,6 _= 33.15, *p *= 0.0012 for SCPI-1 and SCPI-2, respectively) in the SCPI-treated groups at all post feeding time points (Fig. 5B, SCPI-1 and -2). The amount of hemolymph [^3^H]-cholesterol at each time point was significantly higher in control larvae than that of SCPI-1-treated larvae (Fig. 5B, control and SCPI-1) whereas there were insignificant differences at the same time point in the amount of [^3^H]-cholesterol in the midgut tissue between the controls and SCPI-1-treated larvae (Fig. 5A, control and SCPI-1). The results indicate that the efflux of absorbed cholesterol from the midgut into the hemolymph was affected in SCPI-treated larvae, suggesting that there was an impairment of [^3^H]-cholesterol transport from the midgut into the hemolymph. On the other hand, the amount of labeled cholesterol in the hemolymph 2 to 6 hours post feeding was 28087.6 and 29564.4 DPM/mg protein for SCPI-1 and SCPI-2 treated larvae, respectively (Fig. 5B), showing that the efflux of cholesterol from the midgut into the hemolymph was similar between SCPI-1 and SCPI-2 treated larvae even though SCPI-2 treated larvae had significantly lower levels of labeled cholesterol in the midgut (Fig. 5A). Therefore, SCPI-1 and SCPI-2 had a similar effect on the efflux of cholesterol despite the differences in their efficiency in inhibiting cholesterol binding to MsSCP2 (Fig. 4).

The insect fat body is the site of cholesterol storage during the feeding stage [38]. The amount of [^3^H]-cholesterol increased 3-fold from 2 hours to 6 hours post feeding in the fat body of the control larvae, indicating an uptake of [^3^H]-cholesterol over time (Fig. 5C, control). The results from the controls are consistent with other studies in which ingested [^3^H]-cholesterol is rapidly accumulated in the fat body in *Manduca *larvae [38]. In contrast, in SCPI-1 or SCPI-2 treated larvae, the accumulation of [^3^H]-cholesterol in the fat body within 6 hours post feeding was significantly lower (*F*_2,9 _= 29.64, *p *= 0.0001) than that of in the controls (Fig. 5C, SCPI-1 and SCPI-2). This is consistent with the observation that the total amount of [^3^H]-cholesterol is significantly lower in the hemolymph of SCPI-treated larvae (*F*_2,9 _= 26.59, *p *= 0.002; Fig. 5B). The 6 hour-post feeding time represented the accumulation of labeled cholesterol stored in the fat body for the observed period. SCPI-1 and SCPI-2 treatment suppressed cholesterol storage in *M. sexta *larvae within 6 hours of treatment by 37% and 69%, respectively (Fig. 5C, 6 hour). SCPI-2-treated larvae had significantly less stored [^3^H]-cholesterol than the SCPI-1 treated larvae (Fig. 5C, 6 hour) although the amount of labeled cholesterol in the hemolymph in both treatments were similar (Fig. 5B, SCPI-1 and SCPI-2). The result implies that SCPI-2 was more effective than SCPI-1 in blocking the uptake of cholesterol from the hemolymph into the fat body. The results of this study provide the evidence for the first time that SCPIs inhibit cholesterol uptake into the midgut, reduce the efflux of absorbed cholesterol into the hemolymph, and block the uptake of cholesterol in the fat body *in vivo *in *M. sexta *larvae.

**Figure 6:**
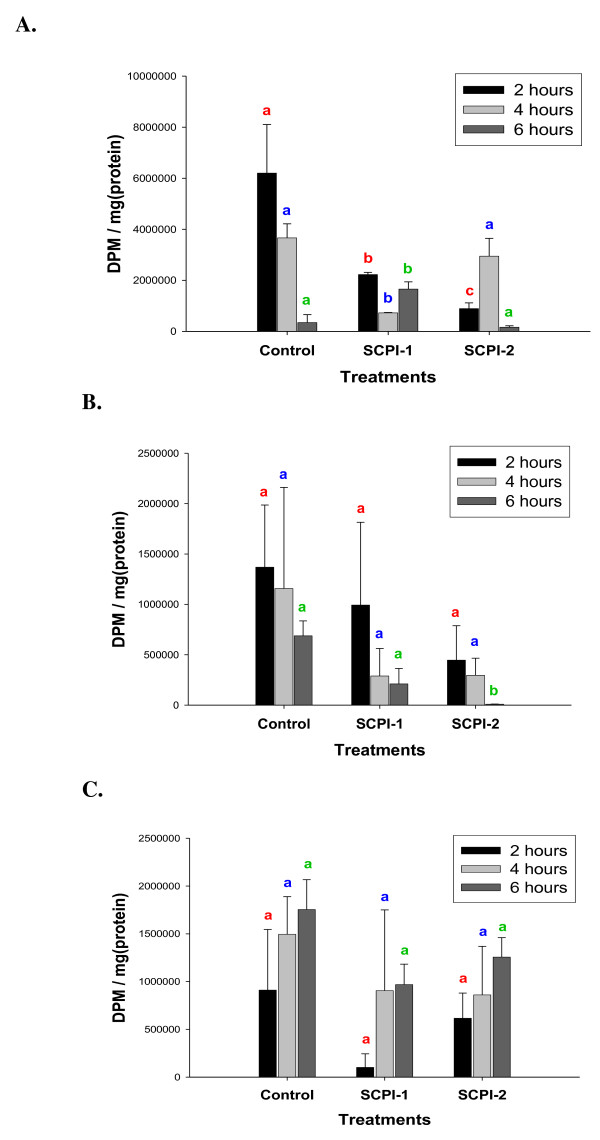
**Effects of SCPIs on [9,10-^3^H]-palmitic acid uptake in *M. sexta *Day 3 4^th ^instar larvae**. **(A) **The midgut. **(B) **The hemolymph. **(C) **The fat body. Values = mean ± S.D. (N = 2). Details of the label/chase experiment are provided in the "Materials and Methods". The same letters above the bars in each time point represent that the mean values did not differ from other group of the same time point significantly (*p *> 0.05) in paired t-tests.

Vertebrate SCP-2 binds to fatty acid and cholesterol [26]. To investigate whether MsSCP-2 is also involved in fatty acid transport in the midgut, we fed labeled [^3^H]-palmatic acid in a label/chase experiment as described above. In the midgut tissue of the control group, there was a significant rapid decrease (*p *= 0.05) in the levels of [^3^H]-palmatic acid from 2 to 6 hours post feeding (Fig. 6A), indicating a very quick clearance of absorbed labeled fatty acid in the midgut. This is consistent with the previous reports that showed rapid uptake and clearance of free fatty acid from the midgut into the hemolymph within 4 hours post feeding [37,39]. In the SCPI-treated larvae, there was a significant reduction of [^3^H]-palmatic acid levels (*F*_2,9 _= 15.15, *p *= 0.0013) in the midgut tissue except at the 4 hour time point in the SCPI-2 treated group (Fig. 6A), suggesting that there was a generalized suppression in the uptake of free fatty acid from the midgut lumen into the midgut tissue in SCPI-treated larvae. However, there were no significant differences between the controls and SCPI-treated larvae in the quantity of [^3^H]-palmatic acid in the hemolymph (Fig. 6B) or in the fat body (Fig. 6C), implying that the clearance of absorbed fatty acid from the midgut and the storage of absorbed fatty acid in the fat body were not affected by SCPI-treatment.

## Discussion

There are multiple steps in the cellular uptake of lipids. This process may be important for insects since receptor or lipoprotein mediated endocytosis and/or exocytosis is not involved in cellular lipid transfer [13]. Lipids can easily diffuse into the outer layer of the cytoplasm membrane where they flip-flop across the membrane bilayer. Cytosolic lipid carriers such as cytosolic fatty acid binding protein and SCP-2 desorbs the free lipids from the cytoplasm membrane and deliver them to the storage site or funnel them into a metabolic pathway [40,41]. The mechanisms underlying MsSCP-2-mediated cellular cholesterol uptake are unclear. AeSCP-2 is not a membrane bound protein [31], and MsSCP-2-mediated lipid absorption is unlike membrane transporters, such as Niemann-Pick C1-like 1 (NPC1-L1) [42]. MsSCP-2 may enhance the desorption of membrane-bound ligand through the collision with membrane-bound cholesterol [9], which, in turn, may lead to a MsSCP-2-mediated cholesterol diffusion into the cytosol. The SCP-2/membrane collision model is the proposed mode for fatty acid transport by the SCP-2 of *Yarrowia lipolytica *[43].

In an earlier study, sterol carrier protein-2 inhibitors (SCPIs) were identified via high throughput screening using recombinant AeSCP-2 [34]. AeSCP-2 is not a product of the AeSCP-x gene [18]. However, both AeSCP-x and AeSCP-2 belong to the SCP-2 gene family [28]. The SCPIs inhibit ligand binding in both AeSCP-2 and the SCP-2 domain protein of AeSCP-x, although the EC_50_s of the same SCPI are higher for the SCP-2 domain protein of AeSCP-x than for AeSCP-2 [34]. SCPIs are lethal to both mosquito and *Manduca sexta *larvae [34]. It was speculated that the *Manduca sexta *has a protein homologous to that of mosquito AeSCP-2, which was inhibited by SCPIs to cause the biological effects in *Manduca *larvae [34]. To further investigate the possible existence of *Manduca sexta *SCP-2 (MsSCP-2), we identified MsSCP-x/SCP-2 via reverse-transcriptase PCR based cloning strategy using degenerate primers designed on the basis of a consensus cDNA sequence within insect SCP-x/SCP-2 genes (Fig. 1). In vertebrates, the SCP-x/SCP-2 gene codes two proteins that share a common 13 kDa SCP-2 domain: 58 kDa SCP-x and 15 kDa pro-SCP-2, which is post-translationally cleaved to 13 kDa SCP-2 [26]. MsSCP-X has the same domain architecture as vertebrate SCP-x. The full-length of the MsSCP-Z/SCP-2 cDNA encodes a protein of 536 amino acids (GQ869536). The amino acid sequence similarities between MsSCP-2 and the SCP-2 domain of SCP-x in other insect species were high (≥60%, Table 1).

Using two pairs of primers that detect the N-terminal 2/3-oxoacyl-CoA thiolase domain and the C-terminal SCP-2 domain, it was discovered that the transcripts for MsSCP-x and MsSCP-2 were differentially regulated based on the abundance of each in the same samples (Fig. 2A). The differential production of the transcripts was most evident in the midgut and fat body, at which time the midgut had predominantly MsSCP-2 mRNA, whereas the fat body had a much higher level of MsSCP-x transcript (Fig. 2A, lane A). In the cotton leafworm (*S. littoralis*), two transcripts from a single copy of the SCP-x/SCP-2 gene were detected [19]. However, in the silkworm (*B. mori*), alternative splicing seemed to generate two transcripts from a single copy of the SCP-x/SCP-2 gene [27]. The first 46 bps of the 5'end UTR sequence of MsSCP-x is divergent from the MsSCP-x 5' UTR or the sequence corresponding to the SCP-2 domain (Fig. 1, shaded sequence in 5' UTR of the MsSCP-2 cDNA sequence), whereas the identical sequence in the 5' UTR of MsSCP-2 to the SCP-2 domain of the MsSCP-x transcript started at the same position as the splicing site in the *B. mori *SCP-x/SCP-2 gene (Fig. 1, bold letter "g" in MsSCP-2 5' UTR; [27]). Whether the MsSCP-x/SCP-2 gene may produce two transcripts via alternative splicing needs further investigation.

The spatial expression protein files of MsSCP-x are different from those described in *S. littoralis and B. mori*, in which the SCP-x transcript level is higher in the midgut and Malpighian tubules. In contrast, the MsSCP-x mRNA levels were much lower in the midgut than in other tissues in general (Fig. 2A). The spatial transcription profiles of MsSCP-x and MsSCP-2 are similar to the mRNA levels of the vertebrate SCP-2 gene. The vertebrate SCP-x is detected essentially in all tissues, whereas SCP-2 was in highest abundance in the liver and intestine [26]. The MsSCP-2 transcription levels were higher in the midgut and fat body during the feeding stage (Fig. 2A, lanes A to D); the metabolic function of the fat body is similar to that of the vertebrate liver and adipose tissue. However, the insect SCP-x protein appears to be processed post-translationally to produce two proteins: the 2/3-oxoacyl-CoA thiolase and the SCP-2 [18,19], which would render higher protein levels of SCP-2 in tissues transcribing SCP-x at higher levels.

The anti-SCP-2 domain of AeSCP-x antibodies recognized the purified recombinant MsSCP-2 (Fig. 2B, lane 8) and detected a similar molecular weight immune-reactive protein in the midgut, hindgut, and fat body (Fig. 2B). This result coincides with the fact that the insect midgut is the main site for cholesterol and fatty acid uptake [38]. This also showed the expression pattern of MsSCP-x/SCP-2 is similar to other mosquito SCP-x and SCP-2 genes. In *Aedes aegypti*, AeSCP-2 and AeSCP-x were strongly expressed in the foregut and mostly in the midgut during the larval feeding stages [18,20]. In *Aedes aegypti*, *Spodoptera littoralis *and *Bombyx mori*, SCP-xs had high levels of transcription in the midgut during the last larval feeding stages [18,19,27], however, the SCP-2 domain protein is the prominent product due to post-translational processing of the SCP-x protein in the midgut [18,19]. Therefore, regardless of which transcript of the SCP-x/SCP-2 gene is predominant in the midgut, high levels of SCP-2 domain protein may be the end result.

There was 56% less (*p *= 0.03) stored [^3^H]-cholesterol 6 hours post feeding in the fat body in SCPI-2-treated larvae than for the SCPI-1-treatment (Fig. 6C, SCPI-1 and SCPI-2). In *Manduca sexta *GV1 cells, SCPI-1 and SCPI-2 at 10 μM concentrations suppressed cholesterol uptake by 15% and 22%, respectively [34]. The relatively higher binding affinities of SCPI-2 than that of SCPI-1 to insect SCP-2 [34] (Fig. 4) may result in the differences in biological effects between SCPI-1 and SCPI-2 treatments in which SCPI-2 constantly showed higher efficacy in inhibiting cholesterol trafficking *in vivo *(Fig. 6A and 6C). The results were consistent with the earlier observation that SCPI-2 is more lethal than SCPI-1 to *Manduca *larvae [34]. Both SCPI-1 and SCPI-2 effectively inhibited cholesterol uptake (Fig. 6). There was little labeled cholesterol detected in the feces 2 hours post feeding and a significant increase in [^3^H]-cholesterol in the feces over the observed time period in the control group (Additional file 1), although the excretion of labeled fatty acid during the same period was constant (Additional file 2). The underlying mechanism for the slower excretion of cholesterol than that of fatty acid in feces is unknown. However, [^3^H]-cholesterol levels in the feces within the 6 hour period were 265.84% (*F*_1,6 _= 31.30, *p *= 0.0014) and 369.02% (*F*_1,6 _= 11.35, *p *= 0.015) higher in SCPI-1- and SCPI-2 treated larvae, respectively, than in the controls (Additional file 1). The results indicate that unabsorbed dietary cholesterol was excreted via feces.

Studies using the vertebrate model suggest that SCP-2 mediates cholesterol trafficking and metabolism and is involved in fatty acid uptake [26,44]. However, it is unclear whether insect SCP-2 has similar functions in cholesterol and fatty acid trafficking and metabolism. We investigated the function of MsSCP-2 *in vivo *using sterol carrier protein-2 inhibitors (SCPIs) to examine the biological effect of MsSCP-2 impairment on cholesterol and fatty acid uptake in *M. sexta *larvae. Inhibition of MsSCP-2's function by SCPIs resulted in arrested growth and development in surviving larvae, indicated by lower weight gain over time and delayed pupation (data not shown). When *Manduca *larvae were fed LC_50 _concentrations of SCPI-1 and SCPI-2 for a short time (within 6 hours, see "Methods"), there was a significant reduction in the uptake of labeled cholesterol in the midgut and fat body in SCPI-treated larvae compared to the controls (Fig. 5A and 5C). Similar results were obtained when NBD-cholesterol, a fluorescent cholesterol analog, was used as the tracer for cholesterol uptake *in vivo *(Additional file 2). However, the effect of SCPI-treatment on palmatic acid was limited to the suppression of uptake into the midgut (Fig. 6A and 6C). In insects, the mechanism for the uptake of lipids from the diet into the midgut is unclear. It is hypothesized that lipids (free fatty acid and cholesterol) may be transported into the midgut epithelium via specific membrane transporters, and then trafficked intracellularly by carrier proteins toward the basal side of the midgut for uploading onto the Lp [7]. The efficacy of a SCPI in suppressing cholesterol absorption (Fig. 5A and 5C) seemed to correlate with its efficiency in inhibiting ligand-binding in SCP-2 (Fig. 4) [34], suggesting that intracellular transport plays a critical role in cellular uptake of cholesterol. The fact that inhibition of MsSCP-2's function via SCPIs did not completely abolish cholesterol uptake points to the possibility that there may be more than one class of intracellular carrier proteins for cholesterol in insects. Another candidate as an intracellular cholesterol carrier is the steroid acute regulator protein (StAR)-related lipid transfer protein [21,22].

The mode of larvicidal activity of SCPIs against some insect species, including *Aedes aegypti*, *Anopheles gambiae*, *Culex pipiens *and *M. sexta *is unclear [34,45]. The relatively high binding affinities of SCPI-1 and SCPI-2 to insect SCP-2s indicate that SCPIs may inhibit the biological functions of SCP-2 by competing with cholesterol for binding to SCP-2 [34]. SCPI-1 and SCPI-2 suppressed [^3^H]-cholesterol efflux from the midgut to the hemolymph in *M. sexta *larvae within 6 hours of treatment (Fig. 5B), resulting in the reduction of stored [^3^H]-cholesterol by 37% and 69%, respectively (Fig. 5C). Although, SCPI-1 and SCPI-2 also suppressed [^3^H]-palmatic acid uptake in the midgut (Fig. 6A), there was no evidence that SCPI-treatment impaired the efflux of absorbed [^3^H]-palmatic acid from the midgut into the hemolymph (Fig. 6B). One plausible explanation for the lack of SCPI's effect on the efflux of fatty acid into the hemolymph and ultimately storage in the fat body may be the redundant function of fatty acid binding proteins in the midgut and fat body [46]. The results of this study provide evidence that SCPIs inhibit cholesterol uptake/storage in insects *in vivo*. The effect of SCPI-treatment on fatty acid uptake in larvae was limited to the midgut (Fig. 6). Since insects can synthesize fatty acid *de novo *from acetate [47,48], one possible mode of action of SCPIs' larvicidal activities is reduction in cholesterol uptake over time resulting in a deficiency in cholesterol that might lead to a high mortality rate in SCPI-treated *Manduca *larvae [34]. The likely mechanism of SCPIs in inhibiting cholesterol absorption is the decrease in clearance of absorbed cholesterol from the midgut tissue (Fig. 4B), suggesting that MsSCP-2 may be involved in the cellular trafficking of cholesterol in the midgut.

## Conclusions

In this study we tested the hypothesis that MsSCP-2 may be involved in cholesterol uptake and intracellular transportation. Our results show that *Manduca sexta *sterol carrier protein-x and sterol carrier protein-2 (MsSCP-x/SCP-2) have a high degree of homology in the SCP-2 domain to other insects' SCP-2 and recombinant MsSCP-2 bound to NBD-cholesterol with high binding affinity. Transcripts of MsSCP-2 were highly expressed in the larval midgut and fat body. The result suggests that MsSCP-2 may be involved in cholesterol uptake in the midgut, the main site for cholesterol absorption, and intracellular transportation in the fat body, the main site for cholesterol storage. MsSCP-2 may function as a lipid carrier protein *in vivo*.

We have also shown that sterol carrier protein inhibitors (SCPIs) effectively reduced cholesterol uptake and cholesterol accumulation in the midgut and in fat body.

Due to the total dependence of insects on exogenous cholesterol, targeting insect SCP-2 may be a viable approach for the development of new insecticides.

## Methods

### Chemicals

Chemicals and reagents were purchased from Sigma (Sigma, St. Louis, MO, USA), Fisher Scientific (Pittsburgh, PA, USA) and ICN (Costa Mesa, CA, USA) if their origins were not mentioned in the text. [1,2-^3^H (N)]-cholesterol (40 Ci/mMol) and [9,10-^3^H]-palmitic acid (60 Ci/mMol) were purchased from American Radiolabeled Chemicals, Inc. (St. Louis, MO, USA). AeSCP-2 inhibitors, SCPI-1 (N-4-{[4-(3,4-dichlorophenyl)-1,3-thiazol-2-yl]amino}phenyl)aetamide hydrobromide) and SCPI-2 (8-chloro-2-(3-methoxyphenyl)-4,4-dimethyl-4,5-dihydroisothiazol[5,4-c]quinoline-1(2H)-thione), were purchased from ChemBridge Corporation (San Diego, CA, USA) with at least 90% purity. NBD cholesterol was purchased from Molecular probes (Eugene, OR, USA).

### The tobacco hornworm, *Manduca sexta *

* Manduca sexta *eggs were a gift from Dr. Walter G. Goodman, University of Wisconsin-Madison. Larvae were fed a commercial gypsy moth wheat germ diet (ICN Biomedicals, Irvine, CA), and reared at 25°C and 60% relative humidity, under a 16:8 (Light:Dark) cycle. Fresh food was provided every other day. Fourth instars were selected by observing head capsule slippage at the time of the molt from the 3^rd ^instar and were gated by weight (> 0.35 g, but <0.54 g at 24 h 4^th ^instar and > 0.65 g, but <0.85 g at 48 h 4^th ^instar) [49]. Only gate II larvae were used for each set of experiments.

### RNA extraction and cDNA synthesis of the first strand

Total RNA was extracted from the Day 3 4^th ^instar *Manduca sexta *larvae using TRIzol (Invitrogen, USA) according to the manufacturer's instruction. The midgut was dissected in cold Manduca saline solution [50] under a dissecting microscope and homogenized immediately in 1 ml TRIzol reagent. Five micrograms of each RNA sample were further purified using the TURBO DNA-*free *Kit (Ambion, Austin, TX, USA). The corresponding first strand cDNAs were reverse transcribed from 0.5 μg DNA-free total RNA using Reverse Transcription Kit (Invitrogen, USA). The quantity of the RNA samples was determined by UV_260 _absorption with a NanoDrop™ 1000 spectrophotometer (NanoDrop products, Wilmington, DE).

### Molecular cloning of MsSCP-x/SCP-2 gene

Two degenerate primers were designed for cloning based on the consensus partial cDNA sequence of the SCP-2 domain from *Bombyx mori *(BmSCP-2) and *Spodoptera littoralis *(SlSCP-2). MsSCP-CF1: 5'-CAA ATA CAT GAA GAT CCT TGA-3' and MsSCP-CR1: 5'-TCA ATC CTG CCA GCG GCT TG-3' match to the N-terminal and the C-terminal of the SCP-2 domain, respectively (Fig. 1).

The SMART RACE cDNA Amplification Kit (Clontech, Palo Alto, CA) was used for the 5'-RACE and the 3'-RACE with cDNAs made from the midgut of Day 3 4^th ^instars. The PCR products were separated on 1% agarose gel, purified with a QIAquick Gel Extraction Kit (QIAGEN, Valencia, USA), cloned into pCR-Blunt II-TOPO^® ^blunt plasmid (Invitrogen, Carlbad, CA), transformed into the INV 110 *E. Coli *strain (One Shot^® ^competent cells) (Invitrogen, Carlsbad, CA) and plated on LB plates under Kanamycin selection. Plasmid minipreps of seven clones containing inserts were made using a QiaSpin column (QIAGEN, Valencia, CA) and sequenced in an automatic sequencer (ABI 377XL) using BigDye labeling (Amersham Pharmacia Biotech AB, Uppsala, Sweden). Another degenerate primer (xNF: 5'-TTC AAC GAC AGA ACC AAC CC-3') designed based on the consensus cDNA sequences of the 2/3-oxoacyl-CoA thiolase domain from *Bombyx mori *(BmSCP-x) and *Spodoptera littoralis *(SlSCP-x), and gene specific primers (MsSCP-CR2: 5'-AAA CGG GAC CTA GAA CTA GAA CGG-3, and MsSCP-CR3: 5'-AGA ACT AGA ACG GGA CCT TC-3') derived from the partial cDNA sequence of MsSCP-2 were used to obtain the coding region of the MsSCP-x/SCP-2 gene (Fig. 1). Additional gene specific primer (MsSCP-CR4: 5'-TGG CAA GGT GCA CCT CTG-3', MsSCP-CF2: 5'-TAC GGG TTC AAG GTC AGG AAT GGA-3', and MsSCP-CF3: 5'-AAA CCC GAC GTC ACT TTC AC-3') derived from the coding region was synthesized and used for the 5'-and 3'-RACE to obtain the 5'-and 3'-end of the cDNA. All PCR reactions for MsSCP-2 gene amplification were performed as follows: initial denaturing at 95°C for 3 minutes, followed by 30 cycles of denaturing at 94°C for 30 seconds, annealing at 61°C for 30 seconds, and extension at 72°C for 30 seconds with a final extension of 72°C for 2 minutes. The PCR products were cloned, transformed and sequenced as described above.

### Purification of recombinant MsSCP-2

To produce recombinant MsSCP-2 (rMsSCP-2), PCR products of the entire coding region of the MsSCP-2 gene were cloned into the pGEX-4T-2 GST tag vector (Amersham Pharmacia). PCR primers were 5'-ggctggatccc**CCC**GAGGAGTTCAAAG TG-3' (capital letters are coding sequence; bold letter is the first codon of the MsSCP-2 domain; a BamHI site was incorporated for cloning) and 5'-ccggtgaattcga**CTA **CAGTTTGGAGCGG-3' (capital letters are the antisense of the coding sequence; bold letter is the antisense of the stop codon; the EcoRI site was incorporated for cloning). The expression vector was transferred into the INV 110 E. coli strain (One Shot^® ^competent cells) (Invitrogen, Carlsbad, CA) under 100 μg/ml ampicilin selection. Sequence analysis was performed to confirm that the fusion protein was in the frame with the GST. The rMsSCP-2 expression bacteria were incubated in 200 ml Luria-Bertani (LB) medium with 100 μg/ml ampicilin at 37°C overnight, 50 ml overnight bacterial culture was added into 500 ml fresh LB medium with 100 μg/ml ampicilin and grown at 37°C for 2 hours (OD_600 _= 0.8). Then, expression of rMsSCP-2 was induced by the addition of isopropyl-beta-D-thiogalactoside (IPTG) to a final concentration of 0.2 mM and the culture was incubated at 18°C for about 6 hours.

Cells from the 2.5 L LB medium were harvested by centrifuge at 4000×g for 15 minutes, and then the cellular pellets were resuspended in 30 ml of PBS (140 mM NaCl, 10 mM Na_2_HPO_4_, 1.8 mM KH_2_PO_4_, 2.7 mM KCl, pH 7.4) with 5 mM DTT and 2 mM EDTA. Cells were lysed using a sonic dismembrator (model 300, Fisher, USA). The cell lysate was centrifuged at 20000×g for 1 hour to remove cellular debris. The GST/rMsSCP-2 fusion protein was purified on a GST affinity column (10 ml bed volume; Amersham Pharmacia) and the GST tag was cleaved by digesting with 500 units of Thrombin (Amersham Pharmacia) in the column at 4°C overnight. Thrombin was removed from the eluted rMsSCP-2 by passing it through a benzamidine column (Amersham Pharmacia). Purified rMsSCP-2 was concentrated in a Centricon YM-10 device (Amicon) to 1 mg/ml in a phosphate saline buffer (PBS), pH 7.4, stored in PBS at -80°C. Concentration of the protein was determined using UV 280 nm absorbed coefficiency.

### Tissue and stage expression profiles of MsSCP-x/SCP-2

To investigate the transcription profiles of MsSCP-x/SCP-2 in different tissues at different developmental stages, total RNAs from the hemolymph (hemocytes), midgut, hindgut, fat body, muscle and epidermis were extracted from *Manduca sexta *at different stages (Day 1 4^th ^instar, Day 3 4^th ^instar, Day 1 5^th ^instar, Day 4 5^th ^instar, wandering stage, and pupal stage) and homogenized in 1 ml TRIzol reagent (Invitrogen, USA). Genomic DNA contamination in the 5 μg total RNA samples was treated twice with the TURBO DNA-*free*™ kit (Ambion, USA) and the samples were tested via PCR with MsSCP-2 gene specific primers to verify the RNA samples were free of genomic DNA. Reverse transcription was performed using 0.5 μg DNA-free total RNA and a Reverse Transcription Kit (Invitrogen, USA). Two microliters of the first strand cDNA (equivalent to 50 ng total RNA) was used as a template for MsSCP-x, MsSCP-2 and rpS3 amplification. MsSCP-2 gene-specific forward (MsSCP-CF2) and reverse primers (MsSCP-CR2) were used for the PCR reaction (the expected PCR product was 210 bps; Fig. 1). To detect the MsSCP-x transcript, primers corresponding to the partial sequence of the 2/3-oxoacyl-CoA thiolase domain (forward primer: xNF; reverse primer: MsSCP-CR4) were used for the PCR reaction (the expected PCR product was 151 bps; Fig. 1). Ribosomal protein S3 (rpS3) cDNA fragment was amplified with rpS3-F (5'-TTA ATT CCG AGC ACT CCT TG-3') and rpS3-R (5'-GGA GCT GTA CGC TGA GAA AG-3') primers [51] as an internal control. PCR reactions for MsSCP-2 and MsSCP-x gene amplification were performed as follows: initial denaturing at 95°C for 3 minutes, followed by 30 cycles of denaturing at 94°C for 30 seconds, annealing at 61°C for 30 seconds, and extension at 72°C for 30 seconds with a final extension of 72°C for 2 minutes. PCR control reaction for ribosomal protein S3 (rpS3) was performed as follows: initial denaturing at 95°C for 3 minutes, followed by 30 cycles of denaturing at 94°C for 30 seconds, annealing at 50°C for 30 seconds, and extension at 72°C for 60 seconds with a final extension of 72°C for 2 minutes. The PCR products were separated on 1% agarose gel.

### Western blotting analysis of MsSCP-2 expression

Day 1 5^th ^instars were chilled in ice water for 10 minutes; tissues were dissected out in cold *Manduca *saline solution [50] under a dissecting microscope. Isolated tissues were homogenized in *Manduca *saline solution containing a 0.5 mM benzamidine hydrochloride, 2 mM EDTA, 0.5 mM phenylmethylsulfonyl fluoride, 0.1 mM glutathione, and 0.1 mM protease inhibitor cocktail (Sigma). Concentrations of soluble proteins in each tissue sample were determined using a BCA Protein Assay Kit (Pierce, Rockford, IL, USA).

Proteins (20 μg) from each sample were resolved on a 4-20% pre-casted gradient SDS PAGE gel (ISC Bioexpress, Kaysville, UT, USA) and transferred onto a Hybond-C extra membrane (Amersham BioScience, Piscataway, NJ, USA) at 18 volts overnight in Tris-glycine transfer buffer (0.303% Tris base, 1.44% glycine, 20% methanol) as described [52]. The membrane was incubated for 1 hour in a blocking solution (5% BSA, 5% normal goat serum, 0.5% Tween-20 in phosphate saline buffer (PBST), pH7.4) at room temperature, and affinity purified polyclonal anti-SCP-2 domain of AeSCP-x [18] (1:3000 dilution) was added into the blocking solution and incubated another 1 hour. After 4 washes of 15 minutes each with PBST, the blot was incubated with AP (alkaline phophatase)-conjugated goat anti-rabbit IgG (Jackson ImmunoResearch Laboratories, INC., West Grove, PA, USA) at a 1:2000 dilution in PBST for 1 hour at room temperature. After 4 washes of 15 minutes each with PBST, alkaline phosphate substrate solution (100 mM Tris-Cl pH 9.5, 100 mM NaCl, 5 mM MgCl_2_, with 33 μl of NBT and 17 μl of BCIP in 5 ml of solution) was used to visualize the secondary bound antibodies, which were developed within 30 minutes at room temperature.

### Ligand and SCPIs binding in MsSCP-2

MsSCP-2 binding affinity for NBD-cholesterol, an analog of cholesterol, was determined using NBD-cholesterol binding assays. A 1 ml sample of 10 μM recombinant MsSCP-2 was diluted with 10 mM KHPO_4 _buffer (pH 7.4) to various concentrations (1 nM~10 μM). Twenty-five microliters of each concentration of MsSCP-2 solution and the control (without MsSCP-2) were thoroughly mixed with 25 μl of NBD-cholesterol solution (1 μM in KHPO_4 _buffer, pH 7.4) and allowed to equilibrate for 3 minutes to permit stable measurement of the fluorescence signal. NBD-cholesterol fluorescence was excited at 470 nm, and emission was recorded at 530 nm using a fluorescence microplate reader (Model: GeminiXPS, Molecular Devices). Data were fit using a simple, single binding site, non-linear regression model in GraphPad PRISM^® ^software version 4.0 (GraphPad Software Inc., San Diego, CA) using the equation:

Where, K*d *is the binding affinity, X is the NBD-cholesterol concentration (μM), Y is the total binding (fluorescent intensity unit), and B*max *is the maximum specific binding to be fit. AeSCP-2 and GST were used as a positive control and a negative control, respectively. NBD-cholesterol binding assays for the controls were performed the same as described above for recombinant MsSCP-2.

The inhibitory effect of SCPI on cholesterol binding to MsSCP-2 was measured using NBD-cholesterol competition assays. In a 50 μl reaction solution in each well of a 96-well plate purified recombinant MsSCP-2 (5 μM) was incubated with NBD-cholesterol (1.25 μM) in the presence of increased concentrations of a SCPI (0-12.5 μM) in 10 mM KHPO_4 _buffer (pH 7.4) for 5 min. The fluorescence intensity (excitation/emission = 470/530) of NBD-cholesterol was measured. The background control was NBD-cholesterol alone in 10 mM KHPO_4 _buffer (pH 7.4). A separate set of tests was performed using NBD-cholesterol with increasing concentrations of a SCPI to assess whether a SCPI interfered with NBD-cholesterol fluorescence. If a SCPI interfered with NBD-cholesterol fluorescence, the background control was NBD-cholesterol along with the SCPI. The net change in NBD-cholesterol fluorescence intensity was calculated by subtracting the fluorescence of the background control from the NBD-cholesterol-MsSCP-2 complex in the absence or presence of an inhibitor. The data were plotted with the relative NBD-cholesterol fluorescent intensity (bound NBD-cholesterol) as the y-axis and the molarities of the inhibitor as the x-axis. Bacterial GST protein and *Aedes aegypti *SCP-2 protein (AeSCP-2) were used as a negative control and a positive control for the assays, respectively.

### Effects of SCPIs on lipids uptake

To examine the effect of SCPIs on lipid absorption/trafficking (label/chase), gate II third day 4^th ^instar *M. sexta *were fasted for 2 hr, and then were fed a small piece of diet (0.1 g) containing SCPIs at LD_50 _dosage [34] in 2 μl of ethanol and [^3^H]-cholesterol (0.033 μCi/ml ethanol) or 2 ul of [^3^H]-palmatic acid. For the control group, a small piece of diet (0.1 g) containing only 2 μl of ethanol and [^3^H]-cholesterol (0.033 μCi/ml ethanol) or [^3^H]-palmatic acid was provided to the larvae. Larvae that consumed the diet completely within 30 minutes were selected for the following experiments in three groups (control, SCPI-1 and SCPI-2) of 6 insects (2 insects/time). From the control and treated groups, hemolymph, the midgut tissue, the fat body, and feces from each individual larva were collected at 2, 4 and 6 hr after feeding. The larvae were anesthetized on ice for 5 minutes, the hemolymph was collected via a small cut at the base of the horn and the hemolymph was bled into a clear test tube by gently pressing the larvae body. The entire midgut was dissected out in cold Manduca saline solution [50] containing 0.5 mM benzamidine hydrochloride, 2 mM EDTA, 0.5 mM phenylmethylsulfonyl fluoride, 0.1 mM glutathione, and 0.1 mM protease inhibitors cocktail (Sigma). The dissected midgut was washed in cold PBS to clean off the food content. The fat body was carefully dissected and washed extensively with cold PBS buffer. Collected tissue samples were stored at -80°C. Frozen samples were thawed and homogenized with micropestle in 1 ml of n-hexane:2-propanol (*v:v *3:2). Total lipids including [^3^H]-cholesterol or [^3^H]-palmatic acid were extracted from the midgut, hemolymph, fat body, and feces, using 1 ml of n-hexane:2-propanol (*v:v *3:2). Extracts were centrifuged at 2500 rpm to pellet the denatured protein and other cellular debris. The organic phase containing [^3^H]-lipid was decanted and air dried, then the radioactivity was measured in a 5 ml high flash-point LSC-cocktail (PerkinElmer, Waltham, MA) using a liquid scintillation analyzer TriCarb 2500TR (Packard, Meriden, CT, USA). The denatured protein pellet was dried overnight at room temperature to evaporate the residual organic solvent. The dried protein from the extracts was dissolved overnight in 0.2 M KOH at 65°C and the protein concentration was determined using a BCA Protein Assay Kit (Pierce, Rockford, IL). For the tissue samples, radioactivity and protein content were quantified and expressed as [^3^H]-lipid (DPM)/mg soluble protein. For the feces sample, the total radioactivity was directly quantified.

### Statistics

Data were analyzed with two-way ANOVA (GLM procedure) to determine if the control group and SCPIs treated groups differed significantly. Student t-test was used in cases where a pair of treatments was compared to determine the significance of the differences [53].

## Abbreviations

NBD cholesterol: 22-(*N*-(7-nitrobenz-2-oxa-1,3-diazol-4-yl)amino)-23,24-bisnor-5-cholen-3β-ol; SCP-x: sterol carrier protein 2/3-oxyacyl-CoA thiolase; SCP-2: sterol carrier protein-2; SCPI: sterol carrier protein-2 inhibitor; Lp: lipophorin.

## Authors' contributions

MK and QL designed the study and wrote the manuscript together. MK performed the experiments. Both authors read and approved the final manuscript.

## Supplementary Material

Additional file 1 **Effects of SCPIs on [1,2-^3^H (N)]-cholesterol and [9,10-^3^H]-palmitic acid excretion in *M. sexta *Day 3 4^th ^instar larvae**. Feces were collected during the experiment time period and processed to measure total amount of labeled lipids. **(A) **[^3^H]-Cholesterol. **(B) **[^3^H]-palmatic acid. Values = mean ± S.D. (N = 2). Details of the label/chase experiment are provided in the "Materials and Methods". The same letters above the bars in each time point represent that the mean values did not differ from other group of the same time point significantly (*p *> 0.05) in paired t-tests. Click here for file

Additional file 2 **Dietary uptake of NBD-cholesterol**. NBD-cholesterol and the SCPI were added into the diet and fed to Day 3 4^th ^instar larvae (12 insects (4 insects/time)/group) as described for [^3^H]-cholesterol. Total lipids were extracted from tissue samples as described for [^3^H]-cholesterol. Dried lipids were re-dissolved in 50 μl methanol and the NBD fluorescent was measured (470/530 nm = excitation/emission). RFU = the relative fluorescent unit (sample FU - blank FU). Click here for file
