# Supplementary material for: Sterol carrier protein-x gene and effects of sterol carrier protein-2 inhibitors on lipid uptake in Manduca sexta
Source: BMC Physiol. 2010 Jun 9;10:9. doi: 10.1186/1472-6793-10-9 (PMC2903571; doi:10.1186/1472-6793-10-9)
Supplement: Additional file 1 — Effects of SCPIs on [1,2-3H (N)]-cholesterol and [9,10-3H]-palmitic acid excretion in M. sexta Day 3 4th instar larvae. Feces were collected during the experiment time period and processed to measure total amount of labeled lipids. (A) [3H]-Cholesterol. (B) [3H]-palmatic acid. Values = mean ± S.D. (N = 2). Details of the label/chase experiment are provided in the "Materials and Methods". The same letters above the bars in each time point represent that the mean values did not differ from other group of the same time point significantly (p > 0.05) in paired t-tests. [file 1472-6793-10-9-S1.DOC]

**Additional files**

**Additional file 1. Effects of SCPIs on [1,2-^3^H (N)]-cholesterol and [9,10-^3^H]-palmitic acid excretion in *M. sexta* Day 3 4^th^ instar larvae**

Feces were collected during the experiment time period and processed to measure total amount of labeled lipids. **(A)** [^3^H]-Cholesterol. **(B)** [^3^H]-palmatic acid. Values = mean ± S.D. (N=2). Details of the label/chase experiment are provided in the “Materials and Methods”. The same letters above the bars in each time point represent that the mean values did not differ from other group of the same time point significantly (*p*>0.05) in paired t-tests.

**A.**

**[^3^H]-cholesterol (feces)**

**B.**

**[^3^H]-palmitic acid (feces)**
