# Supplementary material for: Sterol carrier protein-x gene and effects of sterol carrier protein-2 inhibitors on lipid uptake in Manduca sexta
Source: BMC Physiol. 2010 Jun 9;10:9. doi: 10.1186/1472-6793-10-9 (PMC2903571; doi:10.1186/1472-6793-10-9)
Supplement: Additional file 2 — Dietary uptake of NBD-cholesterol. NBD-cholesterol and the SCPI were added into the diet and fed to Day 3 4th instar larvae (12 insects (4 insects/time)/group) as described for [3H]-cholesterol. Total lipids were extracted from tissue samples as described for [3H]-cholesterol. Dried lipids were re-dissolved in 50 μl methanol and the NBD fluorescent was measured (470/530 nm = excitation/emission). RFU = the relative fluorescent unit (sample FU - blank FU). [file 1472-6793-10-9-S2.PDF]

Supplementary Table 1. Dietary uptake of NBD-cholesterol

Tissues	NBD-cholesterol RFU/mg proteins (mean ± S.D.)*				Control vs. treatment**			
	treatment	2 hour	4 hour	6 hour	*F*	df	Residual	*p* value
Midgut	Control	26.46±2.97	26.73±2.82	30.28±5.81	--	--	--	--
	SCPI-1	22.77±3.02	22.61±4.57	17.78±6.43	13.54	1	18	0.0017
	SCPI-2	21.39±2.77	21.45±3.55	17.43±6.46	19.13	1	18	0.0004
Hemolymph	Control	17.04±12.09	21.52±4.35	11.51±4.10	--	--	--	--
	SCPI-1	8.62±3.62	7.04±1.37	5.63±1.62	17.19	1	18	0.006
	SCPI-2	4.93±1.26	4.58±1.47	3.89±0.98	29.61	1	18	<0.0001
Fat body	Control	12.09±3.17	12.03±2.01	13.00±2.17	--	--	--	--
	SCPI-1	8.01±2.84	4.18±1.06	3.75±1.10	61.37	1	18	<0.0001
	SCPI-2	4.20±0.57	7.74±3.56	9.05±4.09	21.42	1	18	0.0002
Feces***	Control	229.32±51.06	80.11±82.28	258.53±130.11	--	--	--	--
	SCPI-1	129.26±20.73	246.15±105.57	347.03±15.50	2.504	1	18	0.131
	SCPI-2	136.07±58.05	429.12±117.29	545.00±100.71	21.95	1	18	0.0002

*NBD-cholesterol and the SCPI were added into the diet and fed to Day3 4th instar larvae (12 insects (4 insects/time)/group) as described for $[^3H]$-cholesterol. Total lipids were extracted from tissue samples as described for $[^3H]$-cholesterol. Dried lipids were re-dissolved in 50 ul methanol and the NBD fluorescent was measured (xxx/xxx=excitation/emission). RFU= the relative fluorescent unit (sample FU – blank FU).

**Data were analyzed using two-way ANOVA.

***The total NBD-cholesterol was measured lipid extract of feces.
